# Potentially harmful advantage to athletes: a putative connection between *UGT2B17 *gene deletion polymorphism and renal disorders with prolonged use of anabolic androgenic steroids

**DOI:** 10.1186/1747-597X-5-7

**Published:** 2010-04-29

**Authors:** Nawed Deshmukh, Andrea Petróczi, James Barker, Andrea D Székely, Iltaf Hussain, Declan P Naughton

**Affiliations:** 1 School of Pharmacy and Chemistry, Kingston University, London, UK; 2 School of Life Sciences, Kingston University, London, UK; 3 Department of Anatomy, Histology and Embryology, Semmelweis University of Medicine, Budapest IX, Tüzoltó utca 58, H-1450, Hungary

## Abstract

**Background and objective:**

With prolonged use of anabolic androgenic steroids (AAS), occasional incidents of renal disorders have been observed. Independently, it has also been established that there are considerable inter-individual and inter-ethnic differences, in particular with reference to the uridine diphosphate-glucuronosyltransferase 2B17 (*UGT2B17*) gene, in metabolising these compounds. This report postulates the association of deletion polymorphism in the *UGT2B17 *gene with the occurrence of renal disorders on chronic exposure to AAS.

**Presentation of the hypothesis:**

The major deactivation and elimination pathway of AASs is through glucuronide conjugation, chiefly catalyzed by the *UGT2B17 *enzyme, followed by excretion in urine. Excretion of steroids is affected in individuals with a deletion mutation in the *UGT2B17 *gene. We hypothesize that *UGT2B17 *deficient individuals are more vulnerable to developing renal disorders with prolonged use of AAS owing to increases in body mass index and possible direct toxic effects of steroids on the kidneys. Elevated serum levels of biologically active steroids due to inadequate elimination can lead to prolonged muscle build up. An increase in body mass index may cause renal injuries due to sustained elevated glomerular pressure and flow rate.

**Testing the hypothesis:**

In the absence of controlled clinical trials in humans, observational studies can be carried out. Real time PCR with allelic discrimination should be employed to examine the prevalence of different *UGT2B17 *genotypes in patients with impaired renal function and AAS abuse. In individuals with the *UGT2B17 *deletion polymorphism, blood tests, biofluid analyses, urinalysis, and hair analyses following the administration of an anabolic steroid can be used to determine the fate of the substance once in the body.

**Implications of the hypothesis:**

If the hypothesis is upheld, anabolic steroid users with a deletion mutation in the *UGT2B17 *gene may be exposed to an increased risk of developing renal disorders. In the current detecting - sanctioning anti-doping system, athletes motivated by the potential to evade detection owing to their unique genetic make-up could subject themselves to a serious health consequence. More research on AAS metabolism in the presence of *UGT2B17 *gene deletion is required. Benefit - harm evaluations in therapeutic use of anabolic steroids should also consider this potential link between *UGT2B17 *gene deletion polymorphism and renal disorders.

## Background

"The purposes of the World Anti-Doping Code and the World Anti-Doping Program which supports it are: i) to protect the Athletes' fundamental right to participate in doping-free sport and thus promote health, fairness and equality for Athletes worldwide, and ii) to ensure harmonized, coordinated and effective anti-doping programs at the international and national level with regard to detection, deterrence and prevention of doping" [1]. Current doping control is primarily based on enforcement, complemented by deterrence strategies. Meticulously regulated testing protocols, including regulated analytical testing in accredited laboratories and international standards are in place to identify those who violate the anti-doping rules worldwide and to deter others from doing so [2,3]. The use of performance-enhancing substances and methods deemed to be prohibited is an anti-doping rule violation that is established by the presence of a prohibited substance or its metabolites or markers in the athlete's sample, typically urine or blood [1].

Aside from an ongoing ethical debate on various facets of doping [4], including moral reasoning [5] and medical ethics [6-9], recent critical analyses of the current anti-doping approach have claimed that the current anti-doping is not fit for purpose [10-13]. Reasons for this criticism encompassed ethical issues around constant surveillance [14,15], analytical difficulties and costs [16,17], as well as marked inter-individual differences [18-20].

Anabolic androgenic steroids (AASs) are synthetic derivatives of the endogenously produced male sex hormone, testosterone, which exhibits both anabolic (protein synthesizing) and androgenic (masculinising) effects. Steroids are one of the most potent and the most widely used performance-enhancing substances both amongst Olympic athletes [21-23] and also those outside of the auspices of the World Anti-Doping Agency (WADA), such as competitive and recreational body builders, professional players or even non-athlete adolescent boys [24,25]. The use of AASs is widespread particularly amongst athletes because such drugs can improve their performance in sports by accelerating muscle growth, increasing aggressiveness and enhancing a sense of well-being. Chronic use of AASs has been known to cause serious adverse effects such as virilization, feminization, liver disorders, neuropsychiatric disorders, adverse blood lipid profiles (increased LDL and decreased HDL), cardiovascular disorders and renal complications [26-28]. Among these, renal diseases have received less attention, most likely because renal disorders are infrequent among AAS users in comparison to other, more prevalent diseases. Throughout the last decade, the literature sporadically presented cases of severe renal disorders among AASs users, especially with elevated and prolonged use [29-34]. The number of incidents presented is well below the estimated number of AAS users, however, the user profiles described in these case studies do not differ significantly from those AASs abusers who do not develop renal complications. Thus, there may be a connection between deletion mutation in a steroid conjugating enzyme and occurrence of renal diseases with chronic use of AAS.

AASs are commonly excreted in urine mainly as glucuronide conjugates, the formation of which is catalyzed by various uridine diphosphate-glucuronosyltransferase (UGT) enzymes. The UGTs 2B7, 2B15 and 2B17 are found to be the principal enzymes involved in glucuronidation of androgens and their metabolites in humans. Glucuronidation of steroids and their phase I metabolites is an important detoxification and deactivation metabolic pathway which is catalyzed mainly by *UGT2B17 *and to a minor extent by *UGT2B15 *[35-38]. In 2008, a few months before the Beijing Olympics, Schultze and colleagues discovered that the occurrence of deletion polymorphism in the gene coding of *UGT2B17 *enzyme affected the urinary excretion patterns of testosterone [18]. As the current doping testing regime relies on detecting the steroid metabolites in urine, *UGT2B17 *deficient drug abusers are likely to test negative despite the use of the drug. Although the enzyme deficient athletes who are aware of their own genetic profile may benefit from evading doping testing, they may be vulnerable to serious health consequences due to inadequate deactivation and elimination of steroids. The potential consequences of this discovery has triggered the WADA and sport governing bodies to examine their testing regimes.

However, it is inevitable that this phenomenon has significant implications beyond the sporting arena. Juul et al. [39] observed in pubertal boys, that the homozygous deletion in the *UGT2B17 *gene, in line with Schulze et al. [18], affected the urinary excretion pattern of androgen metabolites, but not circulating androgen levels. A deficiency of the *UGT2B17 *enzyme decreases the rate of steroid deactivation and elimination, and owing to this, the bioavailability of steroids may be enhanced to a certain extent. Hence, elevated and prolonged use of AAS may predispose the enzyme deficient individuals to detrimental effects, particularly relating to kidney damage.

Therefore, in this paper we hypothesise that the observed renal disorders among AAS users is connected to the genetic profiles of these users and functional polymorphic deletion of the *UGT2B17 *gene significantly increases the chance of developing kidney complications.

### Inter-individual variations in the frequency of UGT2B17 gene deletion polymorphism

The occurrence of the *UGT2B17 *gene varies amongst individuals of various ethnic groups. Wilson et al. carried out a study on African Americans and the Caucasian population, and found that the occurrence of deletion mutation in the *UGT2B17 *gene was five time more frequent in Caucasians than in African Americans [40].

Several comparative studies of urinary steroid concentrations amongst various ethnic groups have been reported and it has been observed that individuals lacking this enzyme have negligible excretion of steroids. Sjöqvist et al. examined the association of androgen excretion with *UGT2B17 *deletion in a population based study comprising of Korean and Swedish participants [22]. It was found that the absence of the *UGT2B17 *gene was seven times more frequent in Koreans than in the Swedish population. On examining the association between deletion polymorphism and urinary levels of androgens it was revealed that testosterone excretion was 16 times higher in Swedish people compared to the Koreans. These findings indicate the importance of this gene in the excretion of steroids.

Baume et al. reported inter-individual variations in the excretion patterns and kinetics of nandrolone and its metabolites after administration of [^13^C] nandrolone to volunteers and noted the possible natural production of nandrolone and its metabolites [20]. However, it can be postulated that the variations in nandrolone excretion could be due to variations in *UGT2B17 *genotypes amongst individuals.

According to WADA guidelines, if the ratio of concentrations of testosterone to epitestosterone glucuronide in urine is greater than 4, then drug doping is suspected [41]. Since deletion polymorphism in the gene coding for the *UGT2B17 *enzyme affects the urinary level of testosterone, the accuracy of the T/E ratio test is challenged. A study carried out on a heterogeneous group of healthy volunteers with different *UGT2B17 *genotypes (*ins/ins, ins/del *and *del/del*) reported that administration of exogenous testosterone to individuals lacking the *UGT2B17 *gene did not yield T/E values above the population based threshold of 4 for all individuals [18]. This is because the testosterone glucuronide excretion rate in individuals with *del/del *genotype was found to be significantly less than those carrying the *ins/del *and *ins/ins *genotype. No significant effect on epitestosterone excretion was observed. The excretion of unconjugated steroid was found to be a minor elimination pathway even in individuals devoid of the gene. This indicates the possibility of an increase in serum levels of biologically active steroids. It has been reported previously that individuals of Asian ethnicity excrete less testosterone which complements the surveillance that the *del/del *genotype is more frequent amongst the Asian population [18]. Many researchers have observed similar difficulty in testing testosterone abuse pertaining to the *UGT2B17 *deletion, and have suggested making use of genotype based cut-off levels to overcome inter-individual and inter-ethnic variations [42].

These results indicate that a deletion polymorphism in the gene coding is associated with urinary levels of steroid glucuronide conjugates. Owing to an impaired glucuronidation pathway, deactivation and elimination of steroids is reduced. This may result in elevated serum levels of active steroids which can be harmful over a long period of time. Thus, specific groups of individuals who are devoid of the *UGT2B17 *gene are more susceptible to adverse health conditions due to chronic exposure to elevated doses of steroids, compared to individuals having the gene.

### Concomitant use of drugs

UGTs not only contribute to AAS glucuronidation but they also act as conjugating enzymes for various other pharmaceutical drugs [35,43]. It has been reported that athletes use AASs in combination with other medications which may enhance the AAS effects and decrease the side effects associated with such performance-enhancing drugs. Co-administration of steroids along with other UGT substrates may lead to competitive inhibition of steroid glucuronidation. Sten et al. discovered that commonly used over-the-counter (OTC) non steroidal anti-inflammatory drugs (NSAIDs) like diclofenac and ibuprofen inhibited the testosterone glucuronidation activity of *UGT2B17*, *UGT2B15 *and some other UGTs that have previously shown low but detectable activity [43]. Compared to *UGT2B17*, *UGT2B15 *was found to be more sensitive to both the NSAIDs, particularly ibuprofen. Since, *UGT2B17 *shares 96% homology with *UGT2B15*, in a *UGT2B17 *deficient individual, *UGT2B15 *may be the major contributor to glucuronidation. However, use of steroids together with NSAIDs will competitively inhibit the steroid glucuronidation activity of *UGT2B15*. The inhibitory effect was also found to be dependent on the *UGT2B17 *genotype. Regular use of such painkillers is common amongst athletes in order to overcome the pain associated with extensive exercise regimes [44]. Thus, athletes devoid of the *UGT2B17 *gene are susceptible to having complications in steroid elimination, as well as to other medications administered simultaneously. Also, such drug combinations can have direct deleterious effects on renal health.

### The occurrence of renal disorders among AAS users

Herlitz and colleagues reported a study on a cohort of bodybuilders (white and hispanic) and observed that long term use of anabolic steroids combined with high protein intake was associated with proteinuria and secondary focal segmental glomerulosclerosis (FSGS). The pattern of glomerular injury observed, involves scarring of the glomerules and is mediated by elevated glomerular filtration rate (hyperfiltration), glomerular pressure and other adaptive structural-functional responses within the kidneys [29]. One of the bodybuilders with a history of occasional urinary tract infection progressed to end stage renal disease (ESRD) after prolonged use of AASs. It has been reported that non obese individuals with increased body mass index (BMI) owing to elevated muscle mass are susceptible to developing secondary FSGS [29,45]. This indicates the possibility of developing serious renal conditions after prolonged use of steroids by the gene deficient individuals. Many researchers have also reported other adverse renal conditions such as acute kidney injury (AKI), nephropathy, diffuse type 1 membranoproliferative glomerulonephritis and also renal failure owing to long term use of AASs [30,31]. In all these case studies, improvements in renal conditions have been observed in patients who discontinued the use of anabolic steroids. Also, acute renal failure has been reported along with cholestatic liver damage after long term use of AASs [32-34]. Disorders in the liver, the major site for detoxification of steroids and other xenobiotics, can hinder its performance and hence worsen the renal conditions due to elevated level of circulating toxins.

## Presentation of the hypothesis

We hypothesize that with chronic and/or excessive use of AAS, individuals with a deletion polymorphism in the *UGT2B17 *gene (*del/del*) carry an increased risk of developing renal disorders owing to an increase in body mass index and possible direct toxic effects of steroids on the kidneys. Inadequate elimination of the biologically active steroids will lead to elevated serum levels and cause surplus increase in muscle mass. An increase in body mass index may cause renal injuries due to sustained elevated glomerular pressure and flow rate.

### The fate of anabolic steroids in UGT2B17 deficient individuals

Once in the body, plasma protein binding and enzymatic conversions of AASs regulate the availability of free and active steroids at local target sites. Depending on the chemical structure, steroids are deactivated primarily in the liver by phase I (e.g., oxidation, reduction, or hydroxylation) and phase II (glucuronide or sulphate conjugation) metabolic reactions [46]. Glucuronidation by UGTs is considered to be the major deactivation and elimination pathway for steroids and their phase I metabolites. The UGTs act by catalyzing the transfer of glucuronosyl group from the uridine 5'-diphosphoglucuronic acid to the steroid molecule. The resulting glucuronide drug conjugates are less toxic, more polar, and hydrophilic in nature and get easily excreted from the body in urine. The 3 α- and 17 β-OH positions of C-19 steroids and their metabolites are the major sites involved in glucuronide conjugation [35].

*UGT2B17 *catalyses glucuronidation at both oxygens whereas *UGT2B15 *acts only at the 17 β-OH position, hence it is less efficient than *UGT2B17 *in the elimination of steroids. It can be argued that in individuals devoid of the *UGT2B17 *gene, other UGTs like 1A1, 1A3, 1A4, 1A8, 1A9, 1A10, 2B4, 2B7, and 2B15 may contribute to steroid glucuronidation [43,47]. But, less than normal production of steroid glucuronides in individuals devoid of the *UGT2B17*gene indicates inadequate efficiency of other UGTs in eliminating steroids. Alternatively, it may be expected that in order to compensate for impaired glucuronidation activity, steroids may be excreted as sulphate conjugates. However, it was determined by Borts and Bowers that individuals with *del/del UGT2B17 *genotypes did not produce more than normal amounts of steroid sulphate [48,49]. Also, the excretion of unconjugated steroids in urine is considered to be a minor elimination pathway, even in *del/del *individuals [18]. Thus, it can be postulated that lipophilic, unconjugated steroids in the systemic circulation may get distributed into body tissues, get incorporated into the hair by endogenous route or get excreted in sweat [50,51]. Drugs excreted in sweat can further get absorbed by hair via endogenous-exogenous pathways.

Since glucuronidation is the major pathway for deactivation and elimination of steroids, *UGT2B17 *deficiency may increase the serum level of biologically active steroids and their metabolites. Thus, it can be postulated that in *UGT2B17 *deficient individuals, chronic exposure to AAS can result in high BMI and circulating levels of proteins, relative to individuals with a functional enzyme. Increase in body mass can force the kidneys to elevate the glomerular filtration rate (hyperfiltration) and glomerular pressure. Adaptive responses to sustained hyperfiltration and elevated glomerular capillary pressures can lead to the development of renal injuries like scarring of glomerules, which may be associated with proteinuria due to elevated circulating proteins. Hence, we postulate that *UGT2B17 *gene homogeneous polymorphism (*del/del*) increases the chance of developing renal disorders with prolonged use of AAS due to increase in BMI and direct toxic effect of steroids on kidneys.

## Testing the hypothesis

As the hypothesis is based on the dysergistic effect of the *UGT2B17 *polymorphism and long term exposure to AAS, a single clinical trial approach does not present a feasible way to generate evidence to support or refute the hypothesis. Therefore an alternative, multi-component approach is proposed with three distinct components: i) delineation of single dose pharmacokinetics, ii) animal model studies, and iii) genotype testing in humans suffering from renal failure.

To delineate single dose pharmacokinetics for commonly abused AAS, a population based study can be carried out which involves the administration of a single dose of a [^13^C] labelled AASs to healthy volunteers with different *UGT2B17 *genotypes. Routine blood tests, biofluid analyses, urinalysis and hair analyses [50,51] will ascertain the deviation in the pharmacokinetics of steroids owing to the prevalence of different *UGT2B17 *genotypes. Thus, the circulating levels of active steroids (plus phase I metabolites) and alternative metabolic pathways of steroids in individuals with impaired glucuronidation activity can be determined.

An animal study involving chronic administration of AASs to mice or rats with different *UGT2B17 *genotypes, accompanied with routine examination of renal function will help in scrutinizing the relationship between development of renal disorders and deletion polymorphism in the gene. In addition to affording AAS induced renal impairment investigations, animal models will facilitate controlled comparative pharmacokinetic studies of short and long term AAS exposure in mice. In addition, these data will allow comparison to the pharmacokinetics from the human single dose study.

For clinical observations, patients with renal impairment after long term use of anabolic steroids should be analysed for genotype using real time PCR with allelic discrimination to determine the prevalence of *UGT2B17 *deletion genotypes (*del/del, ins/del *and *ins/ins*). Studies would also involve the evaluation of other UGTs which have been reported to play a role in AAS glucuronidation to accurately estimate the impact of each phenotype [52]. Thus, prospective or retrospective genotype analysis of tissues from patients undergoing clinical treatments for renal impairment with admitted steroid abuse are recommended. In addition, depending on the pharmacokinetic results from study i), analysis of hair samples could confirm cases of self-reported chronic AAS abuse.

## Implications of the hypothesis

A system that is primarily based on detection-based deterrence and sanctioning may be easily perceived as a barrier to overcome if a competitive edge can be secured by using performance-enhancing substances or methods. Thus, the current repressive approach to anti-doping inevitably leads to finding ways in detection evasion, with health concerns being secondary. In this 'nothing or all' system, the harm reduction approach that characterizes many drug prevention programmes, cannot be accommodated.

Following the paper by Schulze and colleagues [18] on the effect of *UGT2B17 *gene deletion polymorphism on doping tests, it is likely that this discovery is turned into practice and abused by athletes determined to use AAS. In this scenario, athletes believe that they take the known risk associated with AAS use, but not the risk of being caught. However, if the hypothesis is correct, these athletes expose themselves to an increased risk in developing renal disorders as a consequence of their *UGT2B17 *gene deletion polymorphism.

The harm reduction approach to doping, not yet official but increasingly advocated among doping researchers [13,53], would benefit greatly from an empirical verification of the assumption presented in this paper. The potential danger present in this connection, a controlled clinical trial with volunteers is not feasible but primary care physicians and nephrologists are in a favourable position to advance our knowledge in this area.

Although it has been recognized by now that education based anti-doping prevention is a challenging task [54], having objective information is the *sine qua non *of all efforts relying on informed choice. This is not to advocate the less effective loss-framed messages [55] or fear appeals [56] in preventing AAS use, but rather to draw attention to the fact that inter-individual variations in metabolizing steroids could lead to serious health detriments in individuals even at doses below the problematic supraphysiological doses.

Groups that may benefit from this research are not only AAS users who deliberately take advantage of their unique genetic make-up to evade doping testing, hence will face increased risk of developing renal complications, but include other AAS users, such as recreational users and bodybuilders unaware of the risk, who may inadvertently subject themselves to risks of developing kidney disease. Beyond the athletic arena, administration of AAS should be avoided for patients with nephritic symptoms as it may worsen renal conditions. AAS have been found to be beneficial in improving conditions of individuals with muscle wasting disorders. Researchers have observed improvement in lean body mass of individuals with HIV and chronic obstructive pulmonary disease (COPD) after administration of AAS [27]. Thus, AAS, if not misused, can have therapeutic applications. However, patients that are devoid of the gene will be more vulnerable to serious health consequences. As pharmacokinetic investigations are a key part of the proposed hypothesis testing studies, additional information will be generated on inter-individual differences which may have implications beyond the hypothesis. In such situations, alternative treatment should be employed or a genotype based safe dose should be identified.

## List of abbreviations

AASs: Anabolic androgenic steroids; AKI: Acute kidney injury; BMI: Body mass index; COPD: Chronic obstructive pulmonary disease; ESRD: End stage renal disease; FSGS: Focal segmental glomerulosclerosis; HDL: High density lipoprotein; HIV: Human immunodeficiency virus; LDL: Low density lipoprotein; T/E: Testosterone to epitestosterone ratio; UGT2B15: Uridine diphosphate-glucuronosyltransferase 2B15; UGT2B17: Uridine diphosphate-glucuronosyltransferase 2B17; WADA: World Anti-Doping Agency

## Competing interests

The authors declare that they have no competing interests.

## Authors' contributions

AP conceived the study. All authors have contributed equally to formulating the hypothesis. ND, AP and DPN have drafted the paper. All authors have read and approved the final version of the manuscript.
